# Chronic Visual Abnormality in an Elderly Patient With Temporal Lobe Epilepsy

**DOI:** 10.7759/cureus.56696

**Published:** 2024-03-22

**Authors:** Kiyohiro Atsuji, Shuichiro Neshige, Narumi Ohno, Hirofumi Maruyama

**Affiliations:** 1 Department of Clinical Neuroscience and Therapeutics, Hiroshima University Graduate School of Biomedical and Health Sciences, Hiroshima, JPN

**Keywords:** clinical neurophysiology, loss of awareness, electroencephalography, elderly, focal seizure

## Abstract

A 79-year-old woman visited our department for chronic visual field abnormalities with a floating sensation for two months. Neurological and ophthalmologic examinations yielded normal results, except for brain MRI indicating left hippocampal atrophy. Cognitive function tests were normal. EEG revealed frequent spikes and slow waves in the left frontotemporal region, corroborated by reduced accumulation in ^123^I-iomazenil single photon emission computed tomography. A diagnosis of temporal lobe epilepsy was established, and treatment with lacosamide resulted in a remarkable improvement in symptoms and EEG findings. Mild focal seizures from the temporal region might cause mild impaired awareness, resulting in the patient’s report as a sensation of uncertainty between the self and the outside world, mimicking ophthalmologic abnormalities. The repeated nature of the seizures contributed to the absence of the term "transient" in symptom description. Diagnosing epilepsy in the elderly proves challenging due to nonspecific complaints.

## Introduction

With the rapid aging of society, the prevalence of epilepsy in the elderly is increasing. Temporal lobe epilepsy (TLE) is the most common form in this population. The major seizure type is focal impaired awareness seizure (FIAS). The seizures are accompanied by typical ictal semiology such as motion arrest, dystonia, and oral or hand automatism [1]. Therefore, diagnosis in typical cases of TLE is not difficult. However, diagnosing elderly patients with TLE can be challenging, as the complaints might be unspecific, potentially leading to misdiagnoses such as dementia [2]. Herein, we present an elderly patient finally diagnosed with TLE, whose initial symptom was an ophthalmologic complaint.

## Case presentation

**Figure 1:**
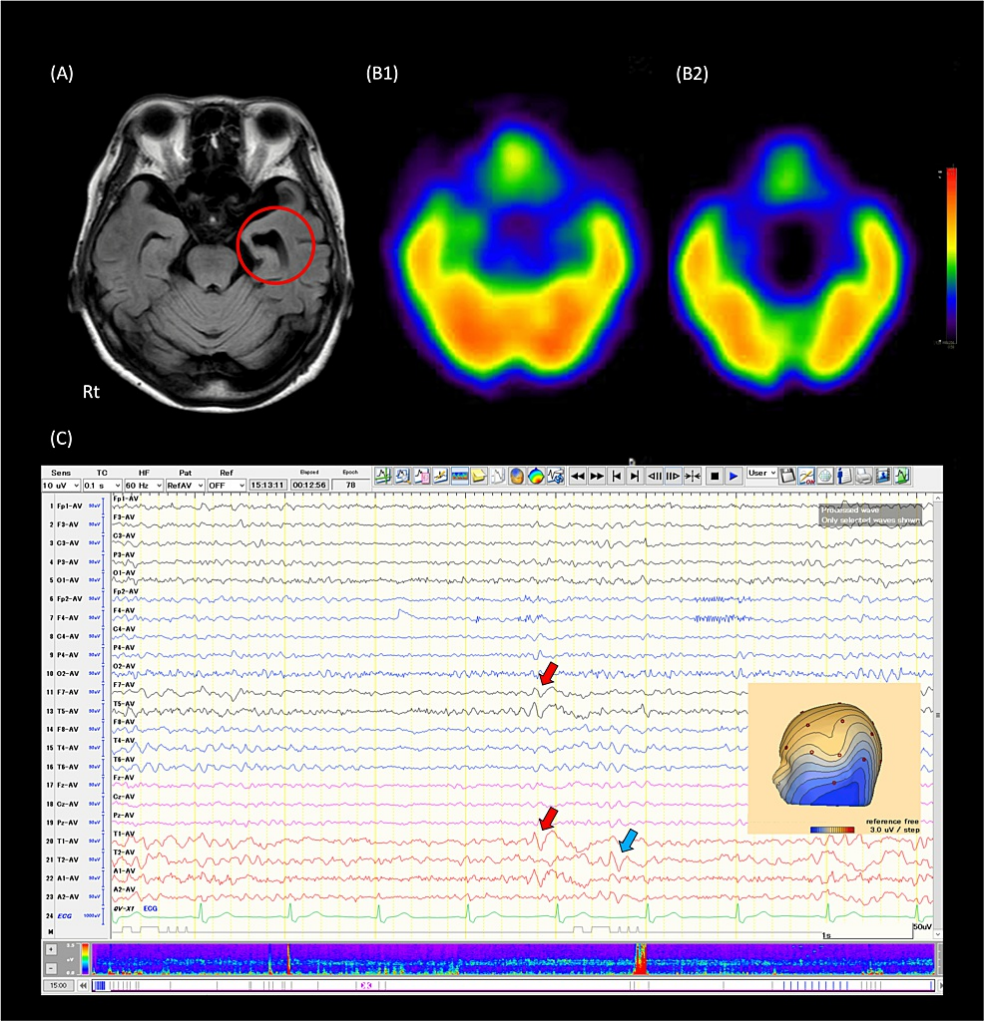
Brain imaging and interictal EEG A. Brain MRI with FLAIR imaging demonstrates atrophy of the medial temporal lobe on the left; B. 123I-iomazenil SPECT (B1: early and B2: late phase) reveals findings compatible with left temporal lobe epilepsy; C. Interictal EEG displays bilateral independent focal sharp waves, predominantly on the left. FLAIR: fluid-attenuated inversion recovery

A 79-year-old woman visited our department for visual chronic field abnormalities with a floating sensation for two months. She complained that her visual symptoms had been persisting. We asked the patient which or all of their visual fields were abnormal, and she responded that her entire visual field was blurred. No visual field abnormalities were elicited when looking in any particular direction. She had a history of well-controlled hypertension and paroxysmal atrial fibrillation managed through oral medication. She had no history of epilepsy and no risk factors for epilepsy such as a precipitating brain injury. The neurological examination yielded normal results. She had no double vision. Repeated ophthalmologic examinations also revealed no abnormalities. Additionally, blood tests revealed unremarkable findings. While brain MRI showed normal findings, including in the optic nerve, it indicated atrophy of the left hippocampus (Figure 1A).

However, cognitive function tests showed normal results (the revised Hasegawa dementia scale was 27/30), and she maintained independence in daily activities. EEG revealed bilateral independent focal sharp waves, occurring every several pages, predominantly on the left, during both awake and drowsy states (Figure 1C). There was no ictal EEG activity. 123I-iomazenil single-photon emission computed tomography (SPECT) demonstrated reduced accumulation in the region (Figure 1B). A diagnosis of temporal lobe epilepsy (TLE) was established, and treatment with lacosamide at a dose of 100 mg/day resulted in a remarkable improvement in both symptoms and EEG findings. She has continued to make regular outpatient visits. Six months have passed without any recurrence of visual field symptoms, and she wishes to continue treatment.

## Discussion

We report a case of an elderly patient with TLE. The diagnostic rationale is supported by the concordance among EEG findings, brain structural findings of MRI (hippocampal atrophy) [3], and findings of brain functional imaging (SPECT) [4]. Additionally, the rapid improvement of symptoms and epileptic findings of EEG following the introduction of anti-seizure medication also supported the diagnosis of the present patient. Therefore, we believe that this condition was consistent with temporal lobe epilepsy in the elderly, even though the patient did not present with seizure-typical symptoms specific to TLE.

It is possible that the patient had a relatively mild degree of FIAS, and thus the degree of impairment was small, and the patient was describing a sensation of uncertainty between the self and the outside world as ophthalmologic abnormalities. Additionally, our case presumably had focal seizures repeatedly. Thus, the patient did not report the symptoms as a "transient" phenomenon by herself, making it challenging for the clinician to include epileptic seizures in the differential at the initial visit.

The MRI of this case was notable in that it showed significant atrophy in the medial temporal lobe, with left-sided predominance. However, it is unlikely that such brain atrophy would occur within a two-month period following the onset of epileptic seizures. Moreover, there were no obvious symptoms of dementia in this case. Thus, it is possible that the long-term repetition of focal seizures, of which the patient was unaware, might have caused chronic brain atrophy.

The clinical course of this case appears to have been somewhat subacute. From this point of view, autoimmune epilepsy enters the differential as the etiology of temporal lobe epilepsy in this case. However, autoimmune epilepsy generally has a more complex seizure pattern and is often associated with impaired consciousness and higher brain dysfunction [5]. Considering the scoring as a diagnostic screening for autoimmune epilepsy [6], it was not necessary to actively consider autoimmune mechanisms in this case.

Epilepsy is most prevalent among the elderly [7]. In Japan, the prevalence of epilepsy in this demographic is on the rise due to the rapid expansion of the geriatric population. Reports indicate that the prevalence of epilepsy in individuals over the age of 65 surpasses 1% of the general population [8]. Approximately half of the initial seizures in the elderly are focal seizures with impaired consciousness, lacking convulsions; about 40% are generalized seizures (evolving from focal to bilateral tonic-clonic seizures); and less than 10% are characterized as generalized seizures such as myoclonic seizures. Older adults often encounter difficulties in accurately describing their symptoms [9]. The variation in seizure types demands close attention. Although epileptic seizures significantly impact the elderly both physically and psychologically, antiepileptic drugs are highly effective when the condition is accurately diagnosed and treated [10]. In Japan's hyper-aged society, comprehending the pathophysiology, diagnosis, and management of epilepsy in elderly patients holds critical importance, both from clinical and social perspectives.

## Conclusions

The number of elderly patients with epilepsy is increasing, and temporal lobe epilepsy is becoming a common disorder in the treatment of the elderly. On the other hand, elderly patients may not present with the typical form of FIAS, or their complaints may be nonspecific, such as visual field abnormalities, as shown in our case. Therefore, clinicians should note that FIAS in elderly-onset TLE may not cause impairment of consciousness. Thus, imaging tests and EEG should be used to further the diagnosis.
